# The Maternal Legacy: Female Identity Predicts Offspring Sex Ratio in the Loggerhead Sea Turtle

**DOI:** 10.1038/srep29237

**Published:** 2016-07-01

**Authors:** Jaymie L. Reneker, Stephanie J. Kamel

**Affiliations:** 1 University of North Carolina Wilmington, Department of Biology and Marine Biology, Center for Marine Science, Wilmington, NC 28403, USA

## Abstract

In organisms with temperature-dependent sex determination, the incubation environment plays a key role in determining offspring sex ratios. Given that global temperatures have warmed approximately 0.6 °C in the last century, it is necessary to consider how organisms will adjust to climate change. To better understand the degree to which mothers influence the sex ratios of their offspring, we use 24 years of nesting data for individual female loggerhead sea turtles (*Caretta caretta*) observed on Bald Head Island, North Carolina. We find that maternal identity is the best predictor of nest sex ratio in univariate and multivariate predictive models. We find significant variability in estimated nest sex ratios among mothers, but a high degree of consistency within mothers, despite substantial spatial and temporal thermal variation. Our results suggest that individual differences in nesting preferences are the main driver behind divergences in nest sex ratios. As such, a female’s ability to plastically adjust her nest sex ratios in response to environmental conditions is constrained, potentially limiting how individuals behaviorally mitigate the effects of environmental change. Given that many loggerhead populations already show female-biased offspring sex ratios, understanding maternal behavioral responses is critical for predicting the future of long-lived species vulnerable to extinction.

Global temperatures are predicted to increase by 0.3–4.8 °C over the next century^1^. Such an increase poses a serious threat for species with temperature-dependent sex determination (TSD), as critical offspring characteristics are determined by the incubation environment^2^. All marine turtles exhibit TSD, where offspring sex is determined by temperatures during the thermosensitive period in the middle third of incubation^3^. Female offspring are produced at higher temperatures and males at lower temperatures within a thermal tolerance range of 25–35 °C^2^. The pivotal temperature, which results in a 1:1 ratio of male to female hatchlings is 29 °C for loggerheads (*Caretta caretta*) in the United States^4^.

The projected increase in global temperatures raises critical concerns for sea turtle populations as warmer nest temperatures have been shown to increase hatchling mortality^5^,^6^. Additionally, even small changes in temperature might dramatically impact offspring sex ratios, since the transitional range of temperatures, where offspring sex shifts from all female (male) to all male (female), can be as narrow as 1 °C^7^, resulting in populations significantly lacking in one sex. Although it has been suggested that female biased sex ratios may increase population growth by providing more nesting females^8^, populations will not be able to withstand continued increases in temperature as mortality and complete feminization of hatchlings will ultimately lead to population extinction by limiting fertilization success and egg production^6^,^9^. Given that the sex ratios of loggerhead nests are already predominantly female-biased for populations studied in Florida, Brazil and the Mediterranean (reviewed in ref. 10), it is important to consider how this species might respond to rapid and, on-going, environmental change.

Theory predicts that the two traits under the greatest selection in the face of increasing global temperatures are (1) the sex ratio reaction norm (*i.e.*, the thermal sensitivity of the sex determination pathway) and (2) maternal nesting behavior, which influences the environment experienced by the developing embryos^11^. It appears that for long-lived species, heritability of the sex ratio reaction norm is too low for this trait to show a strong response to changes in climatic conditions^11^,^12^. Maternal nesting behavior thus becomes the critical trait mediating offspring sex ratios, and its response to climate change will depend on the relative importance of phenotypic plasticity versus genetic adaptation. If mothers can behaviorally alter either their nest placement or timing of nesting (*i.e.*, phenology) to maintain balanced sex ratios in response to local environmental conditions, then this may provide a significant buffer against the effects of rapid environmental change^11^,^13^, and variation among females in the percent of female hatchlings produced should be minimal. Alternatively, mothers could show limited behavioral plasticity, such that nest sex ratios vary little within, but significantly among, females. In this case, an evolutionary response to climate change might be the only possible outcome, with the potential disadvantage that such adaptive changes could occur too slowly in long-lived species^14^.

Here we used data collected for 713 nests from 156 individual loggerhead turtles nesting on Bald Head Island, North Carolina between 1991–2014 (hereafter BHI; Fig. 1) to assess how individual mothers might influence the characteristics of their nests. We used univariate and multivariate models to determine how well maternal identity compared to other spatial, temporal, morphological, and climatic variables in its ability to predict variation in incubation duration, offspring sex ratio (estimated from incubation durations) and hatching success of nests (see Table 1 for all variables). Understanding how the behavior of females in this population is likely to respond to contemporary climate change is especially critical for predicting the future of a species already vulnerable to extinction.

## Results and Discussion

We found that maternal identity was the single best predictor of estimated nest sex ratio, explaining the highest percentage of variation (R^2^ = 0.38) and having a lower AIC score than all other univariate models (Table 2). Additionally, we found significant differences among mothers in their nest sex ratios (ANOVA: *F*_155,557_ = 2.19, P < 0.001; Fig. 2), and a significant repeatability (*r* = 0.22, P < 0.001). We repeated the analyses with incubation duration as the predictor variable and results were nearly identical (ANOVA: *F*_155,557_ = 2.19, P < 0.001; repeatability: *r* = 0.21, P < 0.001; Table 2). This underscores the high degree of consistency within, and significant variability among, females in both their incubation durations and estimated sex ratios.

Given that offspring sex is determined by incubation temperatures during the middle third of incubation, it was not surprising that TEMP was also a significant predictor variable, explaining 32% of the variation in estimated sex ratios (Table 2). Short incubation durations correspond with higher incubation temperatures resulting in the production of more female hatchlings, while the reverse is true for longer incubation durations. Over the 24-year nesting span, average June air temperatures increased significantly from 25.0 °C in 1991 to 26.1 °C in 2014 (r^2^ = 0.17, P = 0.001; NOAA). As such, YEAR was also a significant factor in determining nest sex ratios, explaining 23% of the variation in the univariate model. Air temperatures were also significantly correlated with day of year (r^2^ = 0.35, P < 0.0001), and DOY showed a significant non-linear relationship with estimated sex ratios and incubation duration (Table 2), with male-biased nests produced both earlier and later in the season. Indeed, our multivariate analyses showed that, of the 26 possible model combinations using our five best univariate predictor variables, the model with the lowest AIC score contained TURT, TEMP, MON, YEAR, and DOY and explained 66% of the variation in sex ratio (Supplementary Table S1). However, what is particularly striking is that despite this observed variation in the thermal environment over time, both within and among nesting seasons, individual females still displayed consistent nest sex ratios over the study period. Incubation duration is not simply affected by ambient environmental conditions, but rather is significantly influenced by maternal behavior.

A potential mechanism by which sea turtles could modulate local climatic conditions is through adjustment of nesting phenology^15^. Previous studies have demonstrated an earlier onset of nesting by loggerhead turtles in temperate regions during years with warmer sea surface temperatures^10^,^16^,^17^. To determine whether an individual mother’s nesting phenology was significantly correlated with incubation duration and estimated sex ratio, we performed an analysis of means using the 156 individual turtles. Mean day of year had a significant effect on both mean incubation duration and mean percent female hatchlings (F_2,153_ = 4.34, P = 0.02 and F_2,153_ = 4.28, P = 0.02, respectively; Fig. 3a,b), indicating that mothers nesting at the beginning and end of the season tended to produce clutches with a higher proportion of male hatchlings. If females are consistent in their preference for nesting early *vs.* later in the nesting season, than this might be an important driver of the differences seen among females in incubation duration and estimated sex ratio. However, we did not find significant differences among mothers in their day of nesting (F_155,557_ = 1.16, P = 0.12), and repeatability was not significant (*r* = 0.03, P > 0.05), indicating that, overall, the variability within females in their timing of nesting is substantial, and likely not the main driver of sex ratio differences among females.

**Figure 1:**
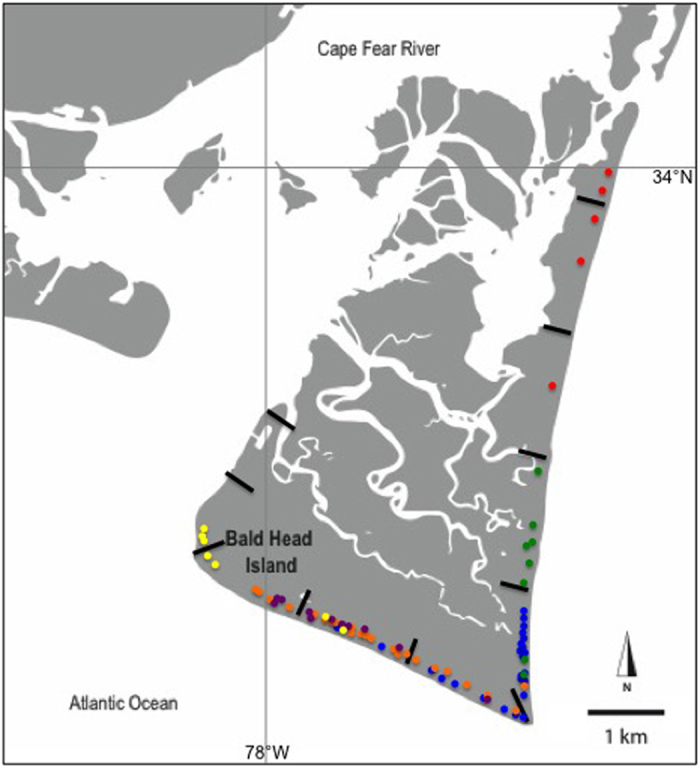
Map of Bald Head Island, North Carolina, USA. Black lines indicate the boundaries of the 10 zones encompassing the west, south and east facing beaches. Each color represents an individual mother (n = 6) with her nest locations represented by dots. Females showed a high degree of beach zone fidelity (*X*^*2*^ = 2336.28, d.f. = 1395, *P* < 0.001). The map was created using Adobe Illustrator CS 6.5 (Adobe Systems Incorporated, San Jose, California) and Microsoft PowerPoint.

**Figure 2:**
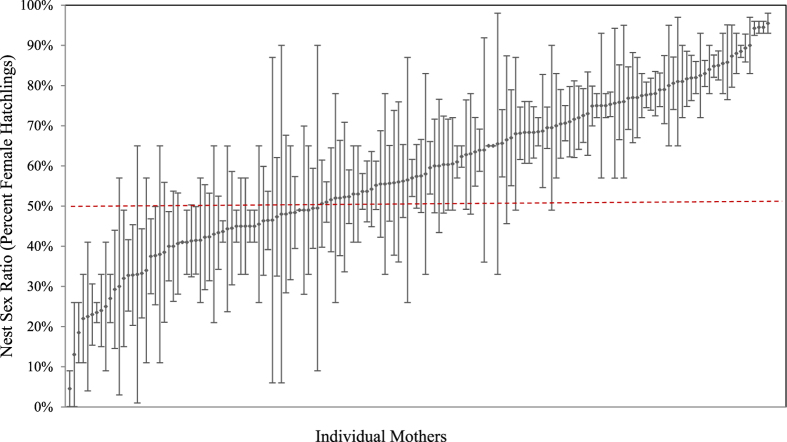
Mean ± SEM of the percent female hatchlings produced in nests of 156 individual females. Each data point represents a female turtle that laid between 2–23 nests, spanning 1–6 nesting seasons. We found significant differences among mothers in their nest sex ratio (*F*_155,557_ = 2.19, P < 0.0001): some mothers consistently produced a high percentage of females, while others consistently produced male offspring. The red dashed line indicates a 50:50 sex ratio.

**Figure 3:**
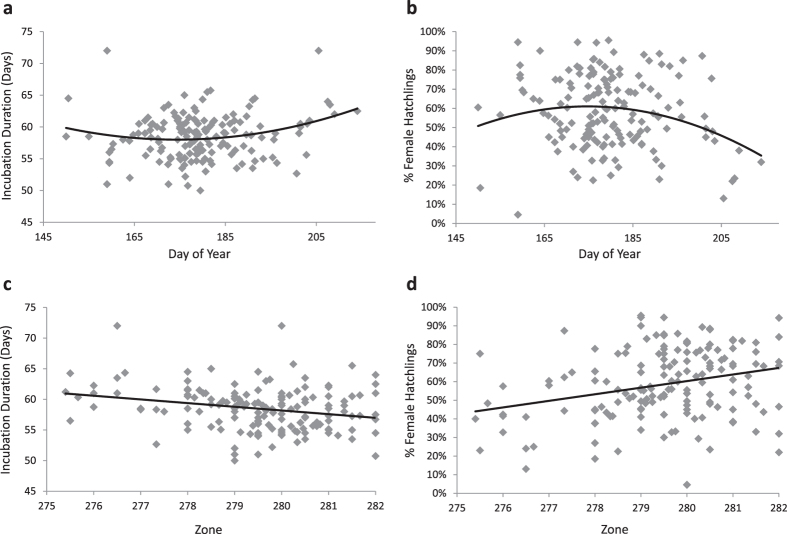
Analysis of means comparing mean day of year for nesting events to (a) mean incubation duration and (b) mean percent female offspring as well as comparing mean beach zone to (c) mean incubation duration and (d) mean percent female hatchlings for all 156 individual mothers, where each data point represents an individual mother. There was a significant relationship between day of year and incubation duration and percent female offspring (r^2^ = 0.05, P = 0.02 and r^2^ = 0.05, P = 0.02, respectively) as well as between beach zone and incubation duration and percent female offspring (r^2^ = 0.06, P = 0.001 and r^2^ = 0.06, P = 0.002, respectively).

Incubation durations also varied spatially at BHI: nests laid on the south beach incubated for 57.6 ± 0.26 days, which was significantly shorter than nests incubating on the east beach (58.9 ± 0.26 days; T-test: *t*_1,707_ = −3.00, P = 0.003). These thermal differences among beaches suggest an additional mechanism by which preferences for particular beach zones might result in consistent differences in incubation durations (and thus sex ratios), as zone significantly predicts percent female offspring (ANOVA: F_9,703_ = 3.32, P < 0.001). We used a contingency test to determine whether females placed their nests non-randomly with respect to the available beach habitat and found that females showed a high degree of beach zone fidelity (*X*^*2*^ = 2336.28, d.f. = 1395, *P* < 0.001; see Fig. 1 for a visual representation of the nests of 6 females). For example, one mother placed 17 of her 24 nests in the same zone over 6 nesting seasons (11 years), with the remaining 7 nests in the two adjacent zones to the south. Though the west beach receives very few nests, likely due to its orientation along the Cape Fear River, the east and south beaches receive similar numbers of nests per year (46.3% vs. 53.2%, respectively; see Methods). As such, beach zone fidelity does not appear to be the result of females’ inability to access certain beach zones. Additionally, an analysis of means comparing mean beach zone preference to incubation duration and estimated sex ratio was significant (F_1,154_ = 10.65, P = 0.001 and F_1,154_ = 9.69, P = 0.002, respectively), suggesting that different beach zone preferences might result in consistent offspring sex ratios within females (Fig. 3c,d). It is important to note that, in the univariate analyses, ZONE accounts for only 9% of the variation in sex ratio, suggesting that mothers are showing even more fine-grained nesting preferences within zones. Previous work on hawksbill turtles (*Eretmochelys imbricata*) found that females in this species show significant preferences for the amount of shade cover above a nest, resulting in distinctive nest sex ratio profiles^18^,^19^. BHI, in contrast, has a relatively homogeneous beach profile with wide, open sandy beaches and no vegetative cover. The beach does slope gradually to the dune ridge, which can vary in severity depending on the tides. Beach slope is thought to play a role in nest-site selection in loggerheads^20^ and might be an environmental cue enabling more fine-grained nest-site discrimination. However, other beach traits, such as sand composition, color, or albedo might also be influencing sand temperatures across beaches. On Ascension Island for example, adjacent beaches have sand temperatures that differ consistently by about 3 °C, a pattern primarily driven by significant differences in sand albedo^21^.

In addition to sand temperature, several features of the nest might indirectly influence incubation durations and offspring sex ratio. Clutch size and hatching success could significantly alter the thermal environment by mediating the degree of metabolic heating (*i.e.* warming within the egg that occurs during embryonic development^22^). Previous studies have shown that metabolic heating during the thermosensitive period is approximately 0.2 °C for loggerhead nests and likely to have a small effect on incubation temperatures^22^,^23^. The highest degree of metabolic heating typically occurs during the final third of development, after hatchling sex has been determined^22^,^23^. We found no significant relationship between clutch size and sex ratio (R^2^ = 0.001, P = 0.47). Hatching success and % female hatchlings were significantly positively correlated, but hatching success only explained 1% of the variation in offspring sex (R^2^ = 0.01; P = 0.003). Finally, we compared mean hatching success and mean percent female offspring for each female using an analysis of means. This relationship was not significant (F_1,154_ = 3.18, P = 0.1), indicating that any potential increase in metabolic heating as a result of a high hatch rate did not affect offspring sex ratio. It is also possible that factors other than nest characteristics are responsible for differences in sex ratios among females. For example, in painted turtles (*Chrysemys picta*), an increase in the endogenous yolk hormone, oestradiol, led to female-biased offspring sex ratios when incubating clutches at a constant temperature^24^. However, studies on the snapping turtle (*Chelydra serpentina*) did not find a relationship between endogenous yolk hormones and offspring sex ratio^25^ and, to our knowledge, evidence of such a relationship in sea turtles is lacking.

In egg-laying species, particularly those which lack parental care, the nest environment influences not only the sex of offspring, but their development and survival as well^26^. However, we found that, here again, maternal identity was the strongest predictor variable, explaining 33% of the variation in hatch rate (Table 2). We also found significant differences among females in their nest success (ANOVA: F_155,557_ = 1.78, P < 0.001): some mothers consistently produced clutches with a high hatch rate, while others deposited clutches with consistently low success rates. The most informative multivariate model contained TURT, along with YEAR, INC DUR, and ZONE (Supplementary Table S1). In contrast to the data on incubation duration, we found no significant relationship between beach zone and hatching success (ANOVA: F_9,703_ = 1.78, P = 0.1), suggesting that nest site location might not be primarily responsible for differences in hatching success. Maternal identity has been shown to significantly influence hatching success in the leatherback sea turtle (*Dermochelys coriacea*), a species known for its very low hatch rates^27^, and different mothers consistently produced clutches with either low or high hatch rates^28^. If hatching success is largely an intrinsic maternal property and fairly robust to environmental variation, this raises important questions about the utility of using nest relocation, a common practice on turtle nesting beaches, to improve hatch rates, especially in light of the often limited resources made available to conservation efforts.

Sea turtle sex ratios have long been a source of great interest, in part due to the common finding of highly female-biased primary sex ratios and the related concern that global climate change may further increase feminization of hatchlings^29^. Given that there is no clear evidence that TSD is adaptive in any long-lived reptile^30^, a balanced sex ratio is likely to be optimal in these organisms. Although recent work has suggested that female-biased hatchling sex ratios may be adaptive in loggerheads as increased breeding periodicity of adult males leads to a more balanced operational sex ratio (OSR; ratio of total breeding males vs females)^31^, the concern remains that continued rapid warming will ultimately lead to a significant reduction of potential male mating partners^32^. As environments vary both in time and space, TSD may thus render populations susceptible to experiencing maladaptive sex-ratio skews, particularly in response to rapidly changing climates.

The data collected over the past 24 years on BHI indicate a high degree of consistency within mothers in the incubation durations of their nests, suggesting that individual plasticity could play a limited role in maintaining balanced sex ratios. Importantly our results contrast sharply with work in other reptiles which suggests that females in these species are able to modify their choice of a nest-site in response to local climatic conditions, providing at least a partial buffer from the effects of climate change^11^,^33^. It appears that for sea turtles, and loggerheads in particular, behavioral plasticity in nest-site choice across spatial and temporal scales is constrained, raising serious concerns about the potential for these animals to compensate for the effects of rapid climate change on time scales relevant to their continued survival.

## Methods

### Study Site

Bald Head Island (33 °N, 78 °W; hereafter BHI) is located in the Cape Fear region of southeastern North Carolina, USA, at the junction of the Cape Fear River and Atlantic Ocean. BHI is designated as terrestrial critical habitat by the United States Fish and Wildlife Service and serves as a high density nesting beach within the northernmost range of the Northwest Atlantic Ocean Distinct Population Segment of loggerhead sea turtles^34^. Though no longer a true island since the closing of an inlet caused by Hurricane Bonnie in 1998, it is now connected to Fort Fisher State Park at its northern tip. BHI has an area of 14.9 km^2^ and is comprised of an extensive salt marsh, dense maritime forest, as well as east-, west-, and south-facing sandy beaches. The beach, which includes the Bald Head State Natural Area, is 16.4 km in length, consists of open sand backed by low-lying dunes and varies in width from 20–55 meters. It is divided into ten 1.6 km zones covering west-, south-, and east-facing beaches (Fig. 1). Since sea turtle nest monitoring began in 1980, BHI has received an average of 96 nests per year ranging from a low of 33 nests in 2014 to a high of 195 nests in 1986. Of the 713 nests used in this study, 46.3% (330/713) were laid on the east beach, 53.2% (379/713) were laid on the south beach and 0.6% (4/713) were laid on the west beach. Loggerheads lay an average of 3–5 clutches (with an average of 100–120 eggs) in one season and return to nest every 2–3 years. Strong beach fidelity seen among females^19^,^35^,^36^ makes it possible to gather extensive data on the same turtle over many nesting seasons.

### Data Collection

Volunteer beach patrollers began monitoring sea turtle nests on BHI in 1980 from May to August. A more formalized monitoring program, managed by the Bald Head Island Conservancy, began in 1991 with the use of metal flipper tags for individual turtle identification (National Band & Tag Company, KY). In 2002, the monitoring project started using passive integrated transponder (PIT) tags (Biomark, ID) in addition to the metal flipper tags. These PIT tags were injected into the triceps muscle complex of the turtle’s shoulder. During the nesting season, trained teams patrolled the beaches nightly from 9 pm–6 am to identify nesting females. When a nesting turtle was encountered, flipper tags were applied to the trailing edges of both fore flippers and morphometric measurements, including curved and straight carapace length, were taken. Measurements were recorded in centimeters to the nearest one hundredth using a caliper and cloth measuring tape. All identified nests were protected from predators such as the red fox (*Vulpes vulpes*), coyote (*Canis latrans*) and raccoon (*Procyon lotor*) by metal cages. Incubation duration was calculated from the night of egg deposition to the night of first emergence of hatchlings. Nests were excavated three days post hatching, or after 70 days of incubation, in order to determine the number of hatched and unhatched eggs. Hatching success was calculated by the number of hatched eggs divided by the total number of eggs in the nest. Methods were carried out in accordance with annual permits authorized by the North Carolina Wildlife Resources Commission. All experimental protocols were approved by the North Carolina Wildlife Resources Commission. We obtained historical daily and monthly air temperatures as well as daily precipitation records from the National Centers for Environmental Information (NOAA) for a nearby weather station in Southport, North Carolina (34 °N, 78 °W) from 1991–2014.

We used the incubation duration of each nest to calculate the percent of female hatchlings within each clutch^37^. Incubation duration is an effective proxy for estimating hatchling sex ratios, particularly in lieu of invasive gonadal sexing techniques, which are required since loggerheads lack sexual dimorphic characteristics as hatchlings^37^,^38^,^39^. Short incubation durations often correspond with higher incubation temperatures resulting in the production of more female hatchlings, while the reverse is true for longer incubation durations^37^ (but see ref. 40 for laboratory data reporting male-hatchlings produced from eggs with shorter than predicted incubation durations). Several studies have highlighted the importance of considering temperature variations during the thermosensitive period for sexual differentiation, pointing out that large deviations from the mean can have important effects on sex determination^41^,^42^. These fluctuations have been shown to be quite dramatic in freshwater turtles which lay shallow nests^41^. However, a high degree of thermal buffering and deeper egg chambers in sea turtle nests tend to dampen the effects of temperature fluctuations^43^. Regardless, multiple laboratory and field studies have validated the use of incubation durations as an effective proxy for sex ratios^37^,^38^,^44^,^45^,^46^. Several factors likely interact to determine offspring sex, however the use of incubation duration, as an indirect measure of temperature, is currently our most effective and non-invasive method for estimating sex ratios^37^.

### Data Analysis

To be included in the analyses, females must have laid multiple nests, either within a season or across multiple seasons. Further, each nest must have a complete data record, including month, day and year laid, beach zone, clutch size, incubation duration and hatching success, as well as maternal straight carapace length and width. In addition, for each nest mean daily precipitation and mean daily air temperatures were averaged over the period of incubation. We used these 12 variables (which all have the potential to influence our response variables) in this analysis with the goal of constructing the most informative model explaining patterns of estimated hatchling sex ratios, incubation duration and hatching success. To narrow down the number of explanatory variables, we first searched for the best univariate models where % female hatchlings, incubation duration and hatching success were regressed against each variable, and compared the explanatory ability of these models using Akaike weights. While these analyses inform our understanding of how individual models compare to one another, we were also interested in combining variables in models to better explain overall variation in our data set. To do this, we built fixed-effects models from the top five predictors in our univariate models, for a total of 26 different models (Supplementary Table S1). Prior to all analyses, we logit transformed % female hatchlings and hatching success to meet assumptions of normality. We compared incubation durations between the south and east facing beaches using a two sample, unpaired t-test. We used a chi-square test with a matrix built from the 10 beach zones and all individual females across their entire nesting record to determine if individual mothers consistently selected the same beach zones for oviposition. We performed an analysis of means comparing individual mothers’ mean hatching success and mean % female hatchling production. We also used an analysis of means to compare the mean day of nesting and mean beach zone with mean incubation duration and mean estimated sex ratio. We calculated the repeatability, which is a measure of the consistency of a trait and often provides an upper limit to estimates of heritability^47^, of offspring sex ratio, incubation duration, hatching success and day of nesting. We used a single-factor ANOVA to compare estimated sex ratio, incubation duration, hatching success, and day of nesting among the 156 individual turtles. We used regression analyses to examine changes in air temperature over the study period. Values are reported as mean ± SE and all analyses were done using JMP Pro 11 (SAS, Cary, NC).

## Additional Information

**How to cite this article**: Reneker, J. L. and Kamel, S. J. The Maternal Legacy: Female Identity Predicts Offspring Sex Ratio in the Loggerhead Sea Turtle. *Sci. Rep.* **6**, 29237; doi: 10.1038/srep29237 (2016).

## Supplementary Material

Supplementary Information

## Figures and Tables

**Table 1:** Variable codes for parameters used in the univariate and multivariate models.

Variable	Code
Turtle ID	TURT
Month Laid	MON
Year Laid	YEAR
Day of Year	DOY
Temperature	TEMP
Precipitation	PRECIP
Location (Zone)	ZONE
Incubation Duration	INC DUR
Hatching Success	HS
Clutch Size	CS
Straight Carapace Length	SCL
Straight Carapace Width	SCW

**Table 2:** Univariate analysis of the relationship between incubation duration, estimated % female hatchlings, and hatching success and various spatial, temporal, morphological, and climatic variables.

Variable	Incubation Duration			% Female Offspring			Hatching Success		
	AIC	p-value	r^2^	AIC	p-value	r^2^	AIC	p-value	r^2^
TURT	1948.22	<0.0001	0.38	338.05	<0.0001	0.38	259.84	<0.0001	0.33
TEMP	1975.32	<0.0001	0.33	404.04	<0.0001	0.32	545.20	0.29	0.00
YEAR	2096.74	<0.0001	0.23	542.61	<0.0001	0.23	468.06	<0.0001	0.10
DOY	2161.77	<0.0001	0.16	600.65	<0.0001	0.16	545.44	0.24	0.00
INC DUR							537.78	0.004	0.01
MON	2213.92	<0.0001	0.10	653.64	<0.0001	0.10	542.68	0.30	0.01
ZONE	2257.03	0.0004	0.09	697.97	0.0006	0.09	530.32	0.07	0.02
PRECIP	2275.73	0.0006	0.02	715.86	0.0006	0.02	534.28	0.001	0.02
HS	2279.12	0.004	0.01	718.77	0.003	0.01			
CS	2287.22	0.50	0.00	727.12	0.47	0.00	540.79	0.02	0.01
SCL	2287.21	0.49	0.00	727.18	0.50	0.00	544.38	0.16	0.00
SCW	2287.66	0.87	0.00	727.63	0.95	0.00	542.21	0.04	0.01

Models are ordered by increasing AIC value for incubation duration and % female offspring; the univariate model with the lowest AIC score for each parameter is indicated in bold. Shaded values represent predictor variables used in multivariate models. DOY is described by adding a quadratic term.
