# Supplementary material for: The Maternal Legacy: Female Identity Predicts Offspring Sex Ratio in the Loggerhead Sea Turtle
Source: Sci Rep. 2016 Jul 1;6:29237. doi: 10.1038/srep29237 (PMC4929680; doi:10.1038/srep29237)
Supplement: Supplementary Information [file srep29237-s1.pdf]

The Maternal Legacy: Female Identity Predicts Offspring Sex Ratio in the Loggerhead Sea Turtle

Jaymie L. Reneker[1,*] and Stephanie J. Kamel[1]

[1]University of North Carolina Wilmington, Center for Marine Science, Department of Biology and Marine Biology, Wilmington, NC 28409, USA

[*] jreneker@gmail.com

Supplementary Table S1. Multivariate analysis of the relationship between (a) incubation duration (b) % female hatchlings and (c) hatching success and various spatial, temporal, and morphological parameters. Models are ordered by increasing AIC value; the multivariate model with the lowest AIC score is indicated in bold. DOY is described by adding a quadratic term.

Model	AIC	p-value	r^2
**(a) Incubation Duration**			
**TURT/MON/YEAR/TEMP/DOY**	**1533.01**	**<0.0001**	**0.66**
TURT/MON/YEAR/TEMP	1539.46	<0.0001	0.65
TURT/YEAR/TEMP/DOY	1588.70	<0.0001	0.63
TURT/MON/YEAR/DOY	1589.27	<0.0001	0.63
TURT/YEAR/TEMP	1606.50	<0.0001	0.62
TURT/MON/TEMP/DOY	1616.28	<0.0001	0.61
TURT/YEAR/DOY	1621.39	<0.0001	0.61
TURT/MON/TEMP	1621.73	<0.0001	0.61
TURT/TEMP/DOY	1667.95	<0.0001	0.58
TURT/TEMP	1688.34	<0.0001	0.57
TURT/MON/YEAR	1704.96	<0.0001	0.56
TURT/MON/DOY	1759.42	<0.0001	0.53
TURT/DOY	1777.20	<0.0001	0.51
MON/YEAR/TEMP	1825.53	<0.0001	0.48
MON/YEAR/TEMP/DOY	1827.34	<0.0001	0.48

TURT/MON	1840.18	<0.0001	0.47
TURT/YEAR	1843.73	<0.0001	0.46
YEAR/TEMP/DOY	1883.99	<0.0001	0.44
YEAR/TEMP	1887.52	<0.0001	0.43
MON/YEAR/DOY	1897.94	<0.0001	0.43
MON/TEMP/DOY	1903.06	<0.0001	0.42
MON/TEMP	1904.63	<0.0001	0.42
YEAR/DOY	1925.80	<0.0001	0.40
TEMP/DOY	1951.64	<0.0001	0.38
MON/YEAR	1986.77	<0.0001	0.35
MON/DOY	2155.34	<0.0001	0.17
**(b) % Female Hatchlings**			
**TURT/MON/YEAR/TEMP/DOY**	**-12.32**	**<0.0001**	**0.65**
TURT/MON/YEAR/TEMP	-6.83	<0.0001	0.65
TURT/YEAR/TEMP/DOY	40.54	<0.0001	0.62
TURT/MON/YEAR/DOY	42.25	<0.0001	0.62
TURT/YEAR/TEMP	57.22	<0.0001	0.61
TURT/MON/TEMP/DOY	65.69	<0.0001	0.61
TURT/MON/TEMP	70.74	<0.0001	0.60
TURT/YEAR/DOY	72.81	<0.0001	0.60
TURT/TEMP/DOY	114.69	<0.0001	0.58
TURT/TEMP	136.95	<0.0001	0.57
TURT/MON/YEAR	152.21	<0.0001	0.56

TURT/MON/DOY	202.73	<0.0001	0.53
TURT/DOY	219.99	<0.0001	0.51
MON/YEAR/TEMP	277.19	<0.0001	0.47
MON/YEAR/TEMP/DOY	279.18	<0.0001	0.47
TURT/MON	280.54	<0.0001	0.47
TURT/YEAR	288.97	<0.0001	0.46
YEAR/TEMP/DOY	333.26	<0.0001	0.43
YEAR/TEMP	336.61	<0.0001	0.42
MON/YEAR/DOY	347.99	<0.0001	0.42
MON/TEMP/DOY	353.85	<0.0001	0.41
MON/TEMP	355.32	<0.0001	0.41
YEAR/DOY	374.41	<0.0001	0.39
TEMP/DOY	399.92	<0.0001	0.37
MON/YEAR	433.75	<0.0001	0.34
MON/DOY	596.51	<0.0001	0.17
**(c) Hatching Success**			
**TURT/YEAR/INCDUR/ZONE**	**191.54**	**<0.0001**	**0.40**
TURT/YEAR/INCDUR/ZONE/PRECIP	192.05	<0.0001	0.40
TURT/YEAR/INCDUR	195.31	<0.0001	0.39
TURT/YEAR/INCDUR/PRECIP	196.43	<0.0001	0.39
TURT/YEAR/ZONE	202.82	<0.0001	0.39
TURT/YEAR/ZONE/PRECIP	203.86	<0.0001	0.39
TURT/YEAR	206.69	<0.0001	0.38

TURT/YEAR/PRECIP	208.25	<0.0001	0.38
TURT/INCDUR/ZONE/PRECIP	241.71	<0.0001	0.35
TURT/INCDUR/ZONE	245.92	<0.0001	0.35
TURT/INCDUR/PRECIP	248.41	<0.0001	0.35
TURT/ZONE/PRECIP	250.54	<0.0001	0.34
TURT/INCDUR	251.05	<0.0001	0.34
TURT/ZONE	254.88	<0.0001	0.34
TURT/PRECIP	257.06	<0.0001	0.34
YEAR/INCDUR/ZONE/PRECIP	448.17	<0.0001	0.14
YEAR/INCDUR/ZONE	449.53	<0.0001	0.13
YEAR/ZONE/PRECIP	455.92	<0.0001	0.12
YEAR/ZONE	456.82	<0.0001	0.12
YEAR/INCDUR/PRECIP	458.63	<0.0001	0.12
YEAR/INCDUR	458.74	<0.0001	0.12
YEAR/PRECIP	468.44	<0.0001	0.11
INCDUR/ZONE/PRECIP	515.75	0.0003	0.05
ZONE/PRECIP	518.58	0.001	0.04
INCDUR/ZONE	525.38	0.01	0.03
INCDUR/PRECIP	529.93	0.0001	0.03
